# Are Female Starlings Able to Recognize the Scent of Their Offspring?

**DOI:** 10.1371/journal.pone.0109505

**Published:** 2014-10-09

**Authors:** Luisa Amo, Gustavo Tomás, Deseada Parejo, Jesús Miguel Avilés

**Affiliations:** Departamento de Ecología Funcional y Evolutiva, Estación Experimental de Zonas Áridas (CSIC), La Cañada de San Urbano, Almería, Spain; Université Lyon, France

## Abstract

**Background:**

Although there is growing evidence that birds may have individual chemical profiles that can function in several social contexts, offspring recognition based on olfactory cues has never been explored. This ability should be more likely evolved in colonial birds and/or species suffering brood parasitism, in which the risk of being engaged in costly misdirected parental care is high.

**Methodology/Principal Findings:**

We performed a choice experiment to examine whether females of the spotless starling, *Sturnus unicolor*, a species that is colonial, and where a fraction of the population is exposed to intraspecific brood parasitism, can discriminate between the scent of their offspring and that of unrelated nestlings. We also explored whether the development of the uropygial gland secretion may play a role in such olfactory discrimination by performing the choice experiments to females rearing nestlings of two different ages, that is, without and with developed uropygial glands. Results showed that female starlings did not preferentially choose the scent of their offspring, independently of whether the gland of nestlings was developed or not.

**Conclusions/Significance:**

Our results suggest that female starlings do not have or do not show the ability to distinguish their offspring based on olfaction, at least up to 12–14 days of nestling age. Further research is needed to examine whether odour-based discrimination may function when fledgling starlings leave the nest and the risk of costly misidentification is likely to increase.

## Introduction

Parental care is costly in terms of energy, time and increased exposure to predation [1]. Therefore, in animals with parental care, natural selection has favoured the evolution of offspring recognition by parents to avoid misdirected parental care. The costs of failing to recognize their own offspring can be particularly high when there are many young conspecifics around, i.e. in colonial birds, or at the moment when fledglings leave the nest and join other young birds forming flocks of juveniles. In these situations, parents should be able to distinguish their own offspring from those of neighboring conspecifics [2]. Therefore, breeding sociality (i.e., being solitary *vs*. colonial) as well as developmental mode and thus the moment when offspring leaves the nest (i.e., altricial *vs*. precocial) are factors that might have modulated the evolution of offspring recognition in animals. This has been illustrated in several avian families such as Laridae [3] or Alcidae [4] that exhibit differences in the moment when parents are able to recognize their offspring in accordance to breeding sociality or age of nestling independence. Similarly, in mammals [5], individual recognition of young associated with exclusive nursing has been demonstrated in “precocial” and colonial species [6].

Another scenario where selection is expected to favor nestling recognition by parents is in species suffering conspecific brood parasitism. Natural selection should have favored the recognition of offspring early in the nestling period, even before fledging, to avoid costs of feeding genetically unrelated conspecific offspring. For example, American coots *Fulica atra*, that show conspecific brood parasitism, are known to learn to recognize and reject parasitic chicks in their brood by using learned cues [7]. However, the mechanisms below such discrimination, if it existed, remain unknown in this as well as in many other bird species challenged by conspecific brood parasites.

During parent-offspring recognition, used cues may be acoustic, visual, olfactory or a combination of them. In birds, individual vocal recognition has been shown as a key component in parent-offspring recognition [8], [9]. In other bird species, however, parents use visual cues to recognize offspring [8]. Also colour signals of offspring quality have been shown to play a role in parent-offspring communication in birds [10]. However, comparative far less attention has been paid to olfactory cues, despite several sources of evidence would suggest they may potentially be involved in offspring recognition in birds.

First of all, olfaction plays an important role in offspring recognition in other taxa with parental care such as mammals [5]. Furthermore, although birds have largely been considered almost anosmic, a growing body of evidence suggests that they can detect odours in different contexts. Indeed, chemical cues affect how bird species interact with their environment and in social contexts (for reviews see [11]–[13]). European rollers *Coracias garrulus* for instance can assess the risk of predation experienced by their offspring based on the smell of an odorous liquid they vomit when scared [14]. Birds can also use chemical cues to locate or identify their nests [15]–[18], and even to discriminate between sexes [19]–[21], and assess the quality of potential rivals [22]. In many instances the source of scent that birds are detecting comes from the uropygial gland secretion ([20], but see [14]), that birds spread on their feathers. This secretion conveys potentially useful information for species recognition [23], as its amount and composition varies [see 24 for a review] between species [23], [25]. Also, within species, the composition and quantity of the secretion can vary between seasons [21], sexes [21], [26], age classes [21], diets [27], [28], hormone levels [29], and individuals [26], [30], [31]. Evidence suggests that this secretion may also inform on genetic compatibility [32], which may be useful during kin recognition [33]–[35] and mate choice. Recent evidence suggests that bird scent is also related to reproductive success [36]. Therefore, it is worth considering the possibility that birds may have evolved the capacity to use chemical cues of the uropygial gland secretion to distinguish their offspring from that of conspecifics. However, to our knowledge, no study has hitherto examined the capability of birds to discriminate between the body odours of their own and foreign nestlings.

In this study we examined whether female spotless starlings *Sturnus unicolor* are able to discriminate between the scent of their offspring and that of foreign nestlings, and whether the uropygial gland secretion of nestlings could play a role in such discrimination. Spotless starlings are an ideal model to cope with our objectives as they are semi-colonial hole-nesting birds that have conspecific brood parasitism [37], [38] and, therefore, natural selection might potentially have favoured discrimination between own and foreign nestlings. In this recognition, use of chemical cues may also be especially useful in species such as the spotless starling that is able to assess the sex of conspecifics by using chemical cues [21]. Also, recent experiments have shown differences in the composition of uropygial gland secretions between 12–14 day-old nestlings and adults [21]. To cope with our objective, we offered females a choice between the scent of one of their nestlings and that of a foreign nestling. Nestlings were matched by age, and choice was tested at two different ages of nestlings, representing two distinct stages of development of the uropygial gland (non-developed vs. completely developed, open and functional). This allowed us to examine the influence of development of the uropygial gland on female odour recognition. If nestling recognition was odour-based in starlings we expected (1) that females could be able to discriminate between the scent of its nestling and that of a foreign nestling; in addition, if the scent was produced by the uropygial gland, we expected (2) that females could discriminate between nestlings once they have developed a completely functional uropygial gland, but not before.

## Results

**Figure 1:**
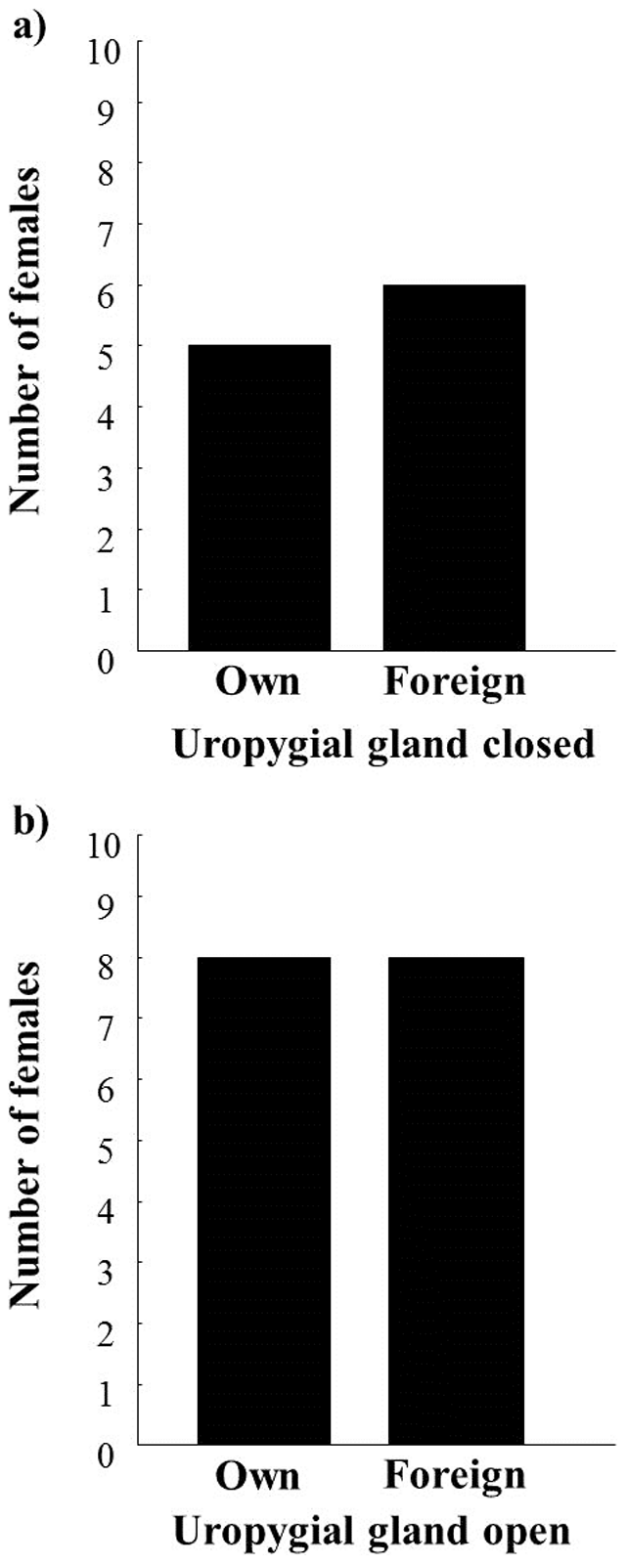
Number of females choosing their own or a foreign nestling scent. Number of female spotless starlings that chose the side of the chamber containing the scent of their own nestling or the scent of a foreign nestling (p = 1.00), when a) nestlings were 5–6 day old and have the uropygial gland closed or b) when nestlings were 12–14 day old and have the uropygial gland open.

When offered the scent of their own *versus* foreign nestlings, a similar number of females chose the scent of their nestling and the scent of a foreign nestling (Wald Stat = 0.00, *p* = 1.00, n =  27), regardless of the development of the uropygial gland (i.e. age of nestlings, Wald Stat = 0.00, *p* = 1.00, n =  27: 11 females with 5–6 day old nestlings and 16 females with 12–14 day old nestlings, Fig. 1). Neither side of the chamber where its own nestling was located (Wald Stat = 0.00, *p* = 1.00, n =  27), nor the fact that females had chosen as soon as the doors were opened or one minute later (Wald Stat = 0.21, *p* = 0.65, n =  27: 5 females chose in the first minute and 21 females after this time) influenced the female choice.

With this sample size and fixing an alpha value of 0.1 (to minimize the probability of incurring in error type II), and a large effect size of 0.25 (to simulate the magnitude estimate of an effect of biological interest), the power of our test is 0.89. Therefore, with a large effect size, we can be fairly confident that our results are not due to low power. On contrast, with an effect size of 0.15, the power of our test is 0.52, and thus, we may not be able to find true choice differences for a medium effect size.

## Discussion

Our results suggest that female starlings cannot discriminate between their nestlings and foreign nestlings by using scent. Females did not choose the side of the chamber containing their offspring. This lack of preference did not depend on the degree of development of the uropygial gland of nestlings; therefore, uropygial gland secretion scent does not seem to be used by females to discriminate between their own nestlings and foreign nestlings.

The absence of odour-based offspring recognition could hardly be explained by a lack of olfactory abilities in starlings because previous results showed that its close relative, the European starling *Sturnus vulgaris* L., can detect chemical compounds in different contexts [39], [40]. More importantly, recent findings suggest that the spotless starling can discriminate the sex of conspecifics based on their scent [21], suggesting that they have the olfactory apparatus needed to discriminate chemicals emitted by their nestlings.

Alternatively, the lack of preference for the own nestling could be explained by a methodological artefact due to the simultaneous presentation of two odours that may hamper the discrimination of scents. This possibility was observed in estrildid finches (*Taeniopygia guttata* and *Lonchura striata var. domestica*) females, whose preference for their own nest scent was masked when they were simultaneously offered their own nest scent with the odour of a foreign conspecific nest [17]. Also, in Leach (*Oceanodroma leucorhoa*) [41] and European (*Hydrobates pelagicus*) storm petrel chicks, the preference for their own nest was hampered when chicks were confronted simultaneously with their own nest odour and a conspecific nest odour, instead of single scents [42]. However, our results are not likely due to a methodological artefact since previous results showing the ability of *S. unicolor* to discriminate the sex of conspecifics where performed using the same apparatus and also presenting two scents simultaneously [21]. Anyhow, we do not know how different is the chemical composition between nestlings of different broods, and therefore, maybe females can simply do not discriminate between their offspring and that of a foreign female because nestling scents do not differ. Chemical analyses need to be performed to try to disentangle whether the variation between broods in the chemical composition of the uropygial gland secretion is enough to allow the evolution of nestling discrimination.

Another possibility to explain the lack of offspring recognition observed in our study is that offspring discrimination is a learned process and thus only expressed later, when nestlings are close to leave the nest (which occurs when nestlings are around 22 days old in this species) and females are able to form clear odour templates of their nestlings. For example, razorbills *Alca torda* do not recognize their own chicks call at 4 days of age whereas they do at 10 days of age [43], and this recognition is exhibited only by males, who takes care of the nestling when it leaves the nest and go to the sea [44]. Similarly, bank swallow *Riparia riparia* parents accepted chicks younger than 16 days-old that were transferred into their nests; rejection of those nestlings began to occur at 16 to 17 days [45]. In a close relative to our study species, the European starling, parents feed foreign nestlings in their nest at least until they are 16 days old but not from 20 days old onwards [46]. However, in this species parents are able to recognize and respond differentially to distress calls of their 15 to 17 days old offspring as compared to other nestlings, outside the nest [47]. Recognition is most developed in species with intermingling of young and where high risk of misidentification exits [45]. Therefore, although offspring recognition may occur even at the nestling stage, parents may not exhibit any discrimination between own and foreign nestlings before fledging time due to the high cost of misimprinting, i.e. to learn to recognize the foreign nestling as the parents' own [48]. Unfortunately, we could not perform a choice experiment with older nestlings due to the high risk they were forced to fledge due to experimenter manipulation.

It is also possible that female spotless starlings rely on mechanisms other than olfaction when discriminating between their own and unrelated nestlings, such as acoustic and/or visual cues [9]. For example, European starling parents are able to discriminate the distress screams of their nestlings from those of unrelated nestlings [47] and to bias food allocation based on color differences between offspring [49]. Alternatively, spotless starlings could rely on olfactory cues in combination with visual or acoustic cues when identifying their offspring. This possibility cannot be ruled out in the system without performing experiments testing the isolated and combined effects of cues from different sensory modalities.

Another alternative explanation to this lack of preference could be that we failed to offer their actual nestlings in the choice tests. As we did not perform parentage analyses before doing the experiment and we do not know the actual rate of conspecific brood parasitism in our population, it is possible that we were challenging some females to choose between two nestlings that are both strange nestlings. In such a case, maybe females did not choose any nestling and this may have masked any preference for the scent of their own nestlings, as olfactory cues have been involved in kin recognition in birds [33]–[35]. Anyhow, this explanation seems unlikely because those nestlings were reared under natural conditions (before and after the experiment) and if females had detected that they were not their offspring, they should not have fed them. This can be explained because the costs of misimprinting are very high due to conspecific brood parasitism, and therefore, natural selection may have favoured females able to recognize and reject parasitic eggs, which would be less costly than rejecting parasitic chicks in case of misimprinting. However, in this species parasitic eggs are rarely rejected once female has begun to lay eggs [38], whereas eggs are rejected by both female and male when introduced before laying [38]. Another explanation is that in our population the risk of intraspecific brood parasitism and/or its cost to hosts is low and therefore, natural selection has not favoured the mechanisms of own offspring recognition. Unfortunately, we do not have data about the rate of intraspecific brood parasitism in this southern population of spotless starlings and therefore we cannot rule out this possibility.

Lack of nestling recognition could also be maintained if costs of recognizing parasitic chicks are higher than benefits of such recognition. In such case, natural selection may have disfavoured the recognition of parasitic nestlings. This could happen if parents do not feed parasitic chicks and their death and subsequent carcass decomposition affect the health of their own chicks. It is also possible if expelling living parasitic chicks from the nest attract predators to the nesting site. In both cases, costs overcome benefits and therefore parents might feed all nestlings, at least as long as they are inside the nest box. It is also possible that in this species, nestlings are who recognize their parents, as they need to know to whom they need to direct their begging signals. For example, zebra finch *Taeniopygia guttata* nestlings are able to recognize the calls of their parents [50] whereas parents do not respond differentially to the distance calls of their own and unrelated nestlings [51], but they do it when exposed to begging calls [52]. Similarly, Herring gull *Larus argentatus* parents are unable to recognize the calls of their nestlings from that of unrelated nestlings, but they recognize them by their behavioural response to parent calls [53]. Therefore, parents may evaluate the behaviour of nestlings towards them, and use this information to recognize their offspring.

Finally, it could be that our study had low power to detect actual differences between nestlings based on olfaction. Thus, with a sample size of 27 females and fixing an alpha value of 0.1 we could have detected large differences in preference (i.e. effect size 0.25; power: 0.89), but not medium differences in the preference (i.e. effect size 0.15; power: 0.52).

Summing up, our results do not support offspring recognition via scent in spotless starling females. However, further research is needed to evaluate whether they use chemical cues to discriminate between their own and unrelated nestlings in a later stage of their development, when nestlings leave the nest and parents need to discriminate between their own and foreign nestlings to provide post-fledging parental care.

## Materials and Methods

### Study species and area

The spotless starling is a medium-size (20–22 cm), hole-nesting passerine that mostly breeds in colonies. Intraspecific parasitism (i.e. egg dumping by other females inside the nest) is common in this species; with around 37% of females suffering from intraspecific parasitism in the first brood and around 20% in the second brood, at least in Central Spain [37]. Incubation takes about 14 days and is predominantly done by females, who also provide most parental care afterwards, although males collaborate to some extent [38]. The nestling period is quite variable, ranging from 18 to 25 days [38].

We performed the experiment in May 2010, when starlings were rearing their nestlings, in a spotless starling population breeding in nest-boxes in Guadix (37°18′ N, 3°11′ W), south-eastern Spain, where starlings breed in colonies of variable size (from 12 to 75 pairs in the studied colonies). We monitored nest-boxes to record hatching dates and we captured 47 female adult starlings with nest-box traps when they were provisioning nestlings of 5–6 day old (N = 31 females) or 12–14 day old (N = 16 females). Starling nestlings at 5–6 day old have their uropygial gland closed (i.e. they do not excrete any substance from their tiny uropygial glands, personal observation), whereas when they are 12–14 day old their uropygial glands are completely developed, open and emit chemical volatiles [21].

After capturing each female, we also captured by hand a randomly selected nestling from her nest and a nestling of the same age from a nearby nest. Starlings, both adults and chicks, were ringed, and introduced in individual clean cotton bags until tested. As soon as the experiment finished, females were released and nestlings were placed back inside their nests. Each female was only used in one trial, so that no female was tested at both nestling ages.

### Experimental design

**Figure 2:**
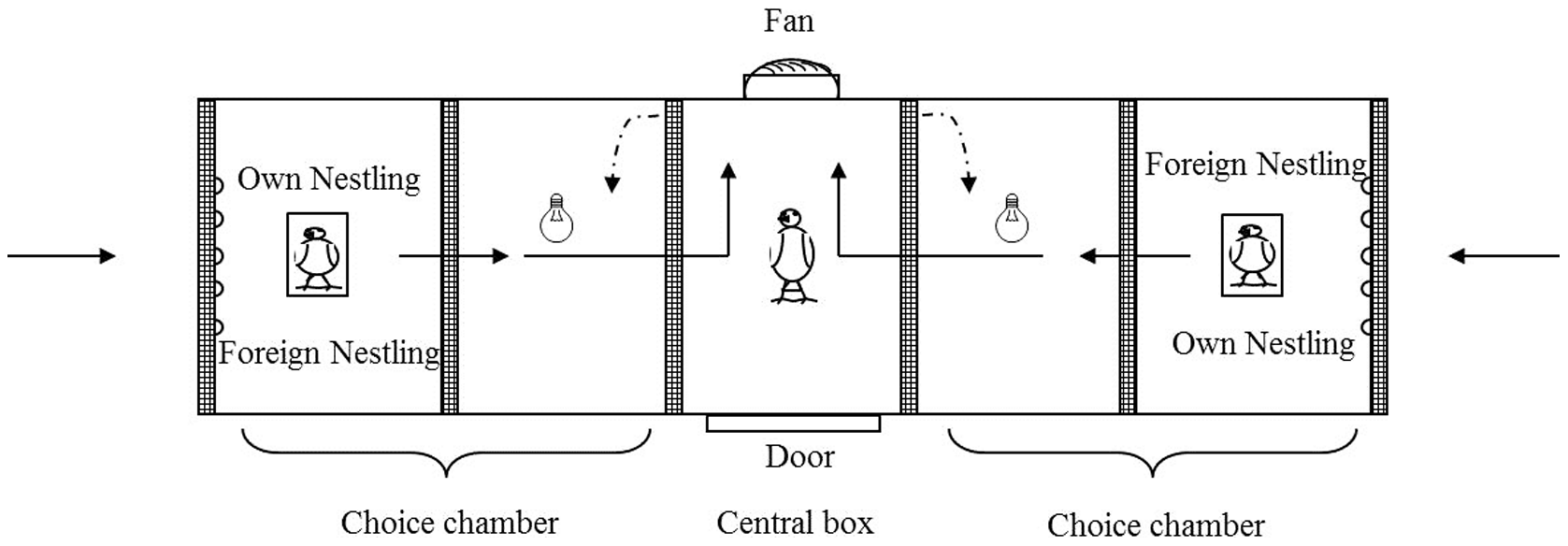
Schematic representation of the olfactometric device used. A focal female was introduced into the central chamber and exposed to scent-carrying air flowing in (solid arrows) from the choice chambers. Odour donor nestlings were hidden from the focal female's view behind a dense plastic mesh (fine cross hatching). A choice was scored when the female entered one of the choice chambers after opening the doors (dotted arrows).

We performed the experiment in an olfactometry chamber in indoor conditions. The device and the methodology have been successfully used in social contexts [21], [22], including determining sex discrimination by scent in adult spotless starlings [21]. The device was composed by a small central plastic box (15×25×25 cm) where the female was introduced. It had a small 12 V PC fan that extracted the air from the device creating a low-noise controlled airflow (Fig. 2). In each test, a female was introduced in the central box and maintained in the dark during 5 minutes. After that, a little lamp (6 V), was lighted in each one of the two choice chambers connected to the central box, and the doors were opened. Each choice chamber was divided into two sectors with screens. The farther sectors of the choice chambers (15×25×25 cm) contained two little plastic recipients (10×4×2) where nestlings were situated. Both, the doors communicating the central chamber with the choice chambers and the screens creating the sectors, were made with a dense plastic mesh that allows air flow but avoids that birds could see through them. The device was hermetically closed and was only opened at the farthest walls of the choice chambers to allow air flow. The fan created two constant air flows, each one entering across the openings located at the farthest walls of each choice chamber, passing through the nestlings and crossing the central chamber, and going outside from the device through the fan (Fig. 2). Thus, the female located in the central chamber received two separate air flows, each one with the scent of the corresponding donor nestling. Donor nestlings were in darkness and in a reduced space, so they could not move. Therefore, the experimental female received the odorous cues of the donor nestlings without any visual cues. The room where the experiment was performed was in complete silence so the experimenter could perceive any noise from any of the birds in the device. In several trials when the nestlings were 5–6 days old (20 out of 31 trials), one or both nestlings emitted calls. These trials were removed from the analysis (final n = 11 females with 5–6 day old nestlings). When nestlings were 12–14 day old, they never called, and therefore, no trials were excluded from the analysis (n = 16 females with 12–14 day old nestlings). The location of own and foreign nestling within the olfactometry device (i.e., left or right side) was randomized between trials. Each pair of nestlings was used twice, one time with each female of each nest. Therefore, the same nestlings were used as scent donor of own nestling and as scent donor of foreign nestling.

We recorded the choice chamber in which the focal female first entered after opening the doors communicating the central chamber with the lateral choice chambers. The use of first choice as a measure of the interest of birds to particular chemical stimuli has been previously validated [21], [22]. In order to minimize the duration of the trials and release the birds as soon as possible to allow females resume the provisioning of nestlings, if after one minute the test female had not left the central chamber, we then gently knocked on the middle of the entry door of the central chamber to stimulate it to move to one of the choice chambers. The knocking on the door did not influence female preference (see Results), in accordance with previous results in this and other species [21], [22]. The olfactometry device was carefully cleaned with 96° alcohol between trials.

### Data analysis

To analyse whether females could discriminate between the scent of their own and foreign nestlings by using chemical cues alone, we performed a generalized linear mixed model with binomial error structure and a logit link function (GLMM). We modelled the probability that a female chose the scent of its own nestling *versus* the scent of a foreign nestling as a dichotomous variable (own nestling (yes) *versus* foreign nestling (no)) in relation to nestling age (5–6 vs 12–14 day old), and taking into account as fixed factors the side of the chamber where the own nestling was placed and whether the experimental bird left the chamber when we opened the doors or after one minute. We included the pair of donor nestlings in the model as a random factor to control for the fact that pairs of donors were used twice. Analyses was performed with the Statistical package R 2.15.1 [54].

### Ethics statement

Birds were healthy during the study and they did not exhibit any sign of stress due to the experiment. Females and nestlings were not kept in captivity more than one hour. Females resumed their provisioning behavior immediately after they were released. No nest abandonment occurred during the experiment or after it, showing a negligible effect of our experiment on starling reproduction. The study was conducted under licence of the Junta de Andalucía, Consejería de Medio Ambiente, Dirección General de Gestión del Medio Natural. The licence covered the capture, measurement and ringing of birds as well as the experimental procedure described above.
